# Soluble immune checkpoint molecules in cancer risk, outcomes prediction, and therapeutic applications

**DOI:** 10.1186/s40364-024-00647-0

**Published:** 2024-09-02

**Authors:** Lin Chen, Yuqing Chao, Wenjing Li, Zhixia Wu, Qinchuan Wang

**Affiliations:** 1 Department of Surgical Oncology, Affiliated Sir Run Run Shaw Hospital, Zhejiang University School of Medicine, Zhejiang, 310009 Zhejiang PR China; 2 School of Public Health, Zhejiang University School of Medicine, Hangzhou, Zhejiang China; 3 Department of Service and Purchase, Affiliated Sir Run Run Shaw Hospital, Zhejiang University School of Medicine, Zhejiang, China

**Keywords:** Soluble immune checkpoint protein, Biomarkers, Cancer immunotherapy, Tumor microenvironment, Cancer risk, Cancer outcomes, Molecular mechanisms

## Abstract

Immunotherapy has emerged as a pivotal modality in cancer treatment, with immune checkpoint inhibitors effectively combating malignancies by impeding crucial pathways within the immune system and stimulating patients’ immune responses. Soluble forms of immune checkpoints exhibit a remarkable diversity and can be readily tracked in circulation, holding immense potential as biomarkers for cancer treatment. An increasing number of studies focused on soluble immune checkpoints in cancer have emerged thanks to technological advancements. In this systematic review, we comprehensively summarized the recent studies on soluble immune checkpoints in human cancer risk prediction, outcome prediction, therapeutic applications, and potential molecular mechanisms, which demonstrated the promising future of soluble immune checkpoints in clinical applications. The clinical relevance of soluble immune checkpoints has been recognized in multiple cancers, yet the therapeutic applications and mechanisms remain obscure. Interpreting the impacts and mechanisms of soluble immune checkpoints could shed a light on the novel strategies of cancer screening, treatments, and outcome prediction.

## Introduction

Immunotherapy has been revolutionizing cancer treatments, especially immune checkpoint inhibitors (ICIs), bringing significant efficacy in solid tumor treatments. Immune checkpoint molecules, like programmed cell death protein 1 and its ligand (PD-1/PD-L1), cytotoxic T-lymphocyte antigen-4 (CTLA-4), could play substantial roles in both maintaining immune tolerance and eliminating tumors. ICIs have been applied in cancer immunotherapy, which demonstrated significant efficacy in multiple types of cancer by activating adaptive immunity.

The efficacy of ICIs mainly depends on the levels of immune checkpoint molecules in tumors [1]. For example, PD-L1 expression on tumor cells has become a common biomarker in selecting lung adenocarcinoma [2] and bladder cancer [3] patients for ICI therapy. However, limited volumes of biopsy samples and intra-tumor heterogeneity restrain the prediction of response to ICIs [4, 5]. Therefore, to identify sensitive, easily acquired and minimally invasive biomarkers is urgently needed.

Soluble immune checkpoint molecules are soluble isoforms of immune checkpoint molecules in circulation, which play distinct roles during carcinogenesis. Soluble immune checkpoint molecules could interact with their receptors/ligands in tumors, thereby affecting anti-tumor immunity. Thus, they have been utilized as biomarkers for predicting disease risks, outcomes, and treatment responses in various cancers. For example, soluble (s)PD-1/PD-L1have a potential role in the prediction of prognosis and treatment of pancreatic cancer (PDCA) [6]. In addition, sPD-L1 and sPD-1 levels could serve as unfavorable prognostic factors for ovarian cancer (OC), and sCTLA-4 is also considered as a potential biomarker in the diagnosis of OC [7]. Despite this, soluble immune checkpoint molecules remain difficult to be detected due to their low levels in the blood. In recent years, multiple technical advances have enabled us to precisely detect the levels of these biomarkers, like sPD-1 and sPD-L1 [8, 9]. Therefore, soluble immune checkpoint molecules could be promising biomarkers in cancer screening, prognosis prediction and treatment.

Here, we systematically review the recent literatures regarding the roles of soluble immune checkpoint molecules in cancer. We elucidate the applications of soluble immune checkpoint molecules in disease risk, outcome prediction and therapeutic potentiality in cancer, reflecting current researches and prospectives. We further illustrate the molecular mechanisms of soluble immune checkpoint molecules in tumor microenvironment (TME), highlighting their crosstalk with key signaling pathways in cancer.

## Soluble immune checkpoint molecules in cancer screening

Soluble immune checkpoint molecules are identified as biomarkers in cancer screening and early detection in multiple cancers. Soluble PD-1, PD-L1, CD28 family of receptors, B7 ligands families, LAG3, etc. all have reported associations with cancer susceptibility. A summary of the information gathered on the soluble immune checkpoints is shown in Table 1.

**Table 1:** The role of soluble immune checkpoint molecules in cancer risk prediction

Soluble receptor/ligand	Risk prediction	The levels in various cancers
sPD-1	↑	• Elevated in the HCC,[10] TNBC,[11] lung adenocarcinoma,[12] aggressive PCa,[13] papillary thyroid cancer,[14] and cHL[15] patients.
	↓	• Decreased in the GC,[16] BC,[17] CRC,[18] RCC[19 and NSCLC[20] patients.
sPD-L1	↑	• Elevated in the SCLC,[21] lung adenocarcinoma,[12] NSCLC,[22] relapsed/refractory multiple myeloma,[23] cartilage bone tumors,[24] aggressive PCa,[13] papillary thyroid cancer,[14] BC,[17] lymphoma,[25–27] cervical cancer,[28] OC,[29] endometrial cancer,[30] mesothelioma,[31] pancreatic cancer,[32] and GC[33 patients.
	↓	• Decreased in the patients with CRC,[18] RCC,[19] BC,[34] and OC.[35]
sPD-L2	↑	• Increased in the NSCLC[36] and pancreatic cancer [32] patients.
sCTLA-4 & sCD28	↑	• sCTLA-4 was elevated in the BCC,[37] GC,[38] and OC[7] patients.
	↓	• sCTLA-4 and sCD28 were decreased in the BC patients [34].
sCD80 & sCD86	↓	• sCD80 and sCD86 levels were significantly lower in the early-stage BC patients compared with the healthy controls [34].
Soluble B7 ligands	↑	• sB7-H4 was increased in the patients with cervical cancer than that in the cervicitis group [39].
	↑	• sB7-H5 levels were increased in the GC, CRC, LC and pancreatic cancer patients [32, 40]
sBTLA	↑	• Upregulated in the PDAC[41] patients.
sHVEM	↑	• Elevated in the patients with GC and BC [42, 43]
	↓	• Decreased in the patients with BC [34]
sLAG-3	↑	• Increased in the pancreatic cancer,[41] advanced ccRCC,[44] and NSCLC[36] patients.
	↓	• Decreased in the patients with BCC,[37] lymphatic leiomyoma,[45] and cervical cancer [46].
sTIM-3	↑	• Increased in the patients with BCC,[37] NSCLC,[36] and PDAC [47].
Soluble TNF	↑	• sCD40 was significantly elevated in the GC[48] and PDAC[49] patients.
	↓	• sCD40L was significantly decreased in the GC[48] patients.
	↑	• sOX40 was increased in the acute T-cell leukemia [50] patients.

Some well-studied soluble immune checkpoints are listed in the above table. ↑ means the soluble immune checkpoints increased in the patients compared with the controls. ↓ means the soluble immune checkpoints decreased in the patients compared with the controls. BC, breast cancer; BCC, basal cell carcinoma; ccRCC, clear cell renal cell carcinoma; cHL, classical Hodgkin lymphoma; CRC, colorectal cancer; GC, gastric cancer; HCC, hepatocellular carcinoma; LC, lung cancer; NSCLC, non-small cell lung cancer; OC, ovarian cancer; PCa, prostate cancer; PDAC, pancreatic ductal adenocarcinoma; RCC, renal cell carcinoma; SCLC, small cell lung cancer; TNBC, triple-negative breast cancer

### Soluble PD-1

PD-1 is the most extensively studied co-inhibitory immune checkpoint receptor in T cells, binding to its ligands PD-L1 and PD-L2. PD-1 could generate soluble isoforms through alternative splicing, which are served as predictive biomarkers in cancer screening.

Elevated sPD-1 levels are significantly associated with increased susceptibility of cancer. A case-control study revealed that with a 1 pg/ml increase in sPD-1 levels, the risk of HBV-associated hepatocellular carcinoma (HCC) increased 2.02-fold in a multivariate logistic regression model [10]. The sPD-1 levels were significantly elevated in triple-negative breast cancer (TNBC) patients before neoadjuvant chemotherapy (NAC) compared to the healthy group (mean ± SD; 549.3 ± 58.76 vs. 379.2 ± 17.30 pg/mL) [11]. Similarly, sPD-1 levels were found significantly higher among the patients compared to the matched healthy donors in lung adenocarcinoma, [11] aggressive prostate cancer (PCa), [12] papillary thyroid cancer, [13] and classical Hodgkin lymphoma (cHL) [14].

However, opposite results were also reported. A case-control study involving 100 gastric cancer (GC) patients and 60 healthy donors found that sPD-1 levels were significantly lower in the former group, while the sPD-1 levels were not associated with cancer risk [15]. Another study showed that the mean levels of sPD-1 were 53.07 ± 24.23 pg/mL in the healthy donors and 47.99 ± 39.32 pg/mL in the group of colorectal cancer (CRC) patients [16]. Similar results were also reported in breast cancer (BC), [17] renal cell carcinoma (RCC), [18] and non-small cell lung cancer (NSCLC) [19].

### Soluble PD-L1

PD-L1 and PD-L2 are major ligands of PD-1, playing substantial roles in ICI therapy. sPD-L1 could also be utilized as a screening biomarker for patients with various cancers, including HCC, GC, lung cancer (LC) and bladder cancer [20].

sPD-L1 has been reported to be associated with disease susceptibility in multiple cancers. In a study of small cell lung cancer (SCLC), the mean sPD-L1 level in the SCLC patients was 1.74 ± 0.82 ng/ml, while its level was 0.59 ± 0.33 ng/ml in the healthy control group [21]. In a prospective cohort study, the preoperative median sPD-L1 levels in the GC patients (71.69 pg/mL) were significantly higher than the healthy controls (35.34 pg/mL), and the area under the curve (AUC) for GC diagnosis based on sPD-L1 was 0.96 (95% confidence interval (CI): 0.93–0.99) [22]. sPD-L1 levels were also found to be significantly elevated in the patients with relapsed/refractory multiple myeloma and bone tumors compared to the healthy donors [23, 24]. Similar results were also observed in studies of aggressive PCa, [12] papillary thyroid cancer, [13] BC, [17] lymphoma, [25–27] cervical cancer, [28] OC, [29] endometrial cancer, [30] mesothelioma, [31] pancreatic cancer, [32] and NSCLC [33].

However, decreased sPD-L1 levels were found in the patients with CRC, [16] RCC, [18] BC, [34] and OC [35]. Heterogeneity among cancer sites, race disparity, retrospective design and different methodologies may influence the findings, a multi-center based prospective study could help address the role of sPD-L1 in cancer.

### The CD28 family of receptors

#### Soluble CTLA-4 and soluble CD28

CTLA-4 competes with CD28 to bind the common ligands B7-1 (CD80) and B7-2 (CD86), constituting the most definitely characterized regulatory T cell pathway [36]. Therefore, CTLA-4 and CD28 soluble isoforms play vital roles in anti-tumor immune responses.

Several studies reported the roles of soluble CTLA-4 and CD28 in cancer screening. One study reported that the median plasma levels of CTLA-4 and CD28 in patients with early-stage BC were both significantly lower than that in the healthy controls [34]. The median sCTLA-4 levels in metastatic melanoma patients were also slightly lower than that in the healthy donors, but the difference was not statistically significant [37]. Interestingly, another case-control study demonstrated that the CTLA-4 levels in patients with basal cell carcinoma (BCC) were significantly increased compared with the healthy individuals, with the AUC of 0.757 (95% CI: 0.597–0.859) for the BCC prediction model [38]. Similar results were also observed in GC [39].

#### Soluble BTLA

B and T lymphocyte attenuator (BTLA) is another substantial co-inhibitory receptor on T cells, and its ligand is herpesvirus entry mediator (HVEM). BTLA/HVEM axis is a promising target for cancer immunotherapy. It was reported that sBTLA levels exhibited a significant increase in the pancreatic ductal adenocarcinoma (PDAC) patients compared to that in the healthy donors. Multivariable logistic regression model indicated that sBTLA was significantly associated with PDAC risk (odds ratio (OR) = 1.46, 95% CI: 1.01–2.17) [40].

### The B7 family of ligands

The B7 family of ligands, belonging to the immunoglobulin superfamily, bind to the CD28 family of receptors on lymphocytes and regulate immune responses through co-stimulatory or co-inhibitory signals [41]. B7 family members, including B7-1/CD80, B7-2/CD86, PD-L2, and B7-H2 play critical roles in cell proliferation, cytokine secretion and TME regulation [42]. A few studies focused on sCD80 and sCD86 found that the median levels of sCD80 (1613.27 vs. 2329.77 pg/mL) and sCD86 (11199.42 vs. 14297.09 pg/mL) were significantly lower in the early-stage BC patients compared with the healthy donors [34].

Interestingly, sB7-H5 levels in the GC, CRC, LC and PDAC patients were significantly increased compared with the healthy controls, which showed a diagnostic value for these cancers [32, 43]. In addition, a retrospective study revealed that sB7-H4 levels gradually increased from cervicitis to cervical cancer, and decreased after treatment [44].

### Soluble LAG-3

Lymphocyte activation gene-3 (LAG-3) is a novel immunosuppressive receptor which is abnormally expressed in various TMEs, and is a substantial immune checkpoint molecule in tumor immune response. sLAG-3 was identified as a promising serum biomarker for the early detection of BCC, [38] and lymphatic leiomyoma [45]. Similarly, Li et al. found that the median sLAG-3 levels in patients with cervical cancer were significantly lower than that in the healthy controls (3.76 vs. 8.36 ng/mL), and low sLAG-3 level was an independent predictor of cervical cancer [46]. However, sLAG-3 was significantly positively associated with PDAC risk (OR = 1.52, 95% CI: 1.04–2.28) in a multivariate logistic regression model [40]. Additionally, increased sLAG-3 levels were also associated with the increased susceptibility in advanced clear cell RCC (ccRCC) [47] and NSCLC [48].

### Soluble TIM-3

T cell immunoglobulin mucin-3 (TIM-3) is a negatively regulated immune checkpoint protein, which inhibits the activation and proliferation of T cells and leads to the immune escape of tumor cells. Therefore, sTIM-3 could be used as a biomarker in cancer screening.

The median levels of sTIM-3 were significantly elevated in BCC patients (7978 pg/mL) compared to the healthy controls (1129 pg/mL), with the AUC of 0.848 (95% CI: 0.721–0.919) in the sTIM-3 incorporated model, suggesting that sTIM-3 could be an effective predictor of BCC susceptibility [38]. Moreover, another study showed that the median sTIM-3 levels were significantly elevated in 45 PDAC patients compared with 50 non-PDAC participants (4585 vs. 2026.5 pg/mL) [47]. Similar results were also found in NSCLC [48]. In addition, sTIM-3, sLAG-3 and sCD137 based signature could help improving the accuracy of NSCLC diagnosis [48].

### The TNF superfamily

The tumor necrosis factor (TNF) superfamily currently comprises 19 ligands and 29 receptors, some of which are expressed on immune cells and participate in the development of tumor-specific immune responses. These molecules also have splicing variants, resulting in soluble isomers that can be traced in body fluids like serum. For example, sGITR, sGITRL, sCD27 and sCD40 were significantly decreased in the patients with early-stage BC [34].

In a prospective and exploratory cohort study, sCD40 levels were significantly elevated in the elderly GC patients compared with the healthy elderly individuals, whereas sCD40L levels were significantly decreased [49]. sCD40 was also considered as a non-invasive biomarker for PDAC diagnosis (AUC = 0.795) [49]. In addition, elevated plasma levels of sOX40 could be used as biomarkers for the diagnosis of acute T-cell leukemia [50].

sHVEM is the soluble isoform of dual immune checkpoint HVEM. One study revealed a significant increase of sHVEM levels in the BC patients (mean ± SD; 4612 ± 2329 vs. 2946 ± 1857 pg/mL) and GC patients (mean ± SD; 4528 ± 1915 vs. 2946 ± 1857 pg/mL) compared to the control group, although this change of sHVEM levels in the CRC patients was not statistically significant [51]. Similar results were obtained in another GC study, where sHVEM levels of GC patients were significantly higher than the non-ulcer dyspepsia patients [52]. By contrast, the early-stage BC patients in another study had relatively lower sHVEM levels compared with the healthy individuals (1866.92 vs. 2290.19 pg/mL) [34].

### Other soluble immune checkpoints

Several soluble immune checkpoint molecules under investigation, like sGARP, sMIC-A, sIDO, sICOS, sCD33, and sTLR-2, showed levels significantly variated in the cancer patients, but their potential of prediction in cancer screening still await further exploration [34, 53, 54, 55, 56, 57].

## Soluble immune checkpoint molecules in cancer outcomes prediction

Soluble immune checkpoint molecules are associated with cancer outcomes, including survival, recurrence, and response to treatment. Understanding the predictive performance of these soluble immune checkpoints on cancer outcomes is conducive to screening the most suitable treatments for patients and monitoring disease development.

### Soluble PD-1

sPD-1 was reported associating with the prognosis of multiple cancers, though the conclusions of some studies remain controversial.

Studies showed that higher baseline sPD-1 levels were associated with poorer prognosis in the patients with diffuse large B cell lymphoma, [58] OC, [7] PDAC, [59, 60] PCa, [55] ccRCC, [18, 61] and CRC [16]. In a multicenter prospective study of 439 GC patients treated with nivolumab, [62] higher sPD-1 levels were associated with the worse overall survival (OS). Melanoma patients with higher baseline sPD-1 levels also experienced the worse OS after ICI therapy [63]. For HCC patients who underwent liver transplantation, Hwang et al. found that higher sPD-1 level was an independent risk factor of recurrence [64]. Patients with TNBC in complete or partial remission to NAC had significantly decreased sPD-1 levels compared to the patients who did not respond well [65]. And increased sPD-1 levels after anti-PD-1 antibody therapy were also found correlating with the accelerated progression of solid tumors [66].

However, other studies demonstrated that sPD-1 could be a favorable prognostic factor for patients with cancers. A Japanese study reported that higher sPD-1 levels were associated with the improved OS in patients with NSCLC receiving anti-PD-1 immunotherapy, [67] which was consistent with another prospective study [68] and a case-control study [69]. Higher sPD-1 levels were also shown to be associated with the better OS in patients with nasopharyngeal carcinoma (NPC) after definitive intensity-modulated radiotherapy, [70] and in GC patients after gastrectomy [71]. In a study of HCC, [72] researchers found that sPD-1 was a favorable independent predictive factor for disease-free survival (DFS) (hazard ratio (HR) = 0.32, 95% CI: 0.14–0.74) and OS (HR = 0.54, 95% CI: 0.30–0.98). Metastatic melanoma patients treated with ICIs were revealed to have increased sPD-1 levels which was correlated to superior progression-free survival (PFS) [73]. And other researchers also found the association between higher baseline sPD-1 levels and better PFS in metastatic ccRCC patients treated with nivolumab [74]. In a study of advanced head & neck cancer (HNC), patients with higher baseline sPD-1 levels responded better to anti-PD-1 treatment than patients with lower concentrations, and these patients experienced prolonged PFS [68].

The intriguing role of sPD-1 in survival prediction of multiple cancers may derived from the interaction between sPD-1 and its ligands in TME. sPD-1 could compete with membrane-bound PD-1 from binding with PD-L1 in vivo, which in turn enhance the anti-tumor immunity [75, 76]. In contrast, sPD-1 could also impair the T cell proliferation and IL2 production through combining with PD-L1 on dendritic cells in vitro [77]. The complicated interaction between PD-1 and its ligands resulted in the alteration of anti-tumor immunity, subsequently affect the survival of cancer patients. However, the mechanisms underlying remain obscure for inconsistent findings between in vivo and in vitro studies, more investigation is warranted to illustrate the mechanisms.

### Soluble PD-L1 and soluble PD-L2

#### Soluble PD-L1

**Fig. 1:**
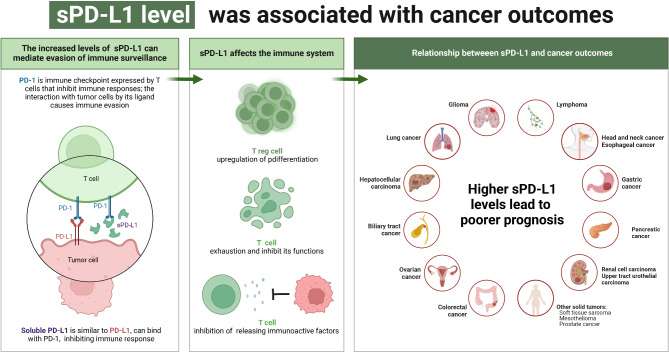
sPD-L1 level was associated with impeded anti-tumor immunity and poor outcomes in multiple cancers. The sPD-L1 could bind with the PD-1 receptor on T cells, thereby inducing T cell exhaustion and inhibiting T cell functions, eventually leading to immune evasion. Elevated levels of sPD-L1 are reported to be associated with the poor outcomes in multiple cancers

As one of the most well-studied soluble immune checkpoint ligands, sPD-L1 was considered an unfavorable prognostic factor in a wide variety of cancers by most studies. To briefly summarize current studies on sPD-L1, we depicted Fig. 1 to show its impact on cancer prognosis.

First, studies revealed that baseline sPD-L1 level was an independent adverse predictor of OS for multiple cancers [78–81]. In prospective studies of NSCLC patients treated with ICIs, patients with higher levels of circulating sPD-L1 had poorer OS [67, 82]. Also, patients with higher sPD-L1 levels had shorter OS than patients with lower levels in a retrospective study of 120 advanced NSCLC patients, [83] and similar conclusions were strongly agreed in several meta-analyses [84–87]. A worse OS was observed in mesothelioma patients with higher baseline sPD-L1 levels [31]. In a cohort of 219 NPC patients, higher sPD-L1 levels appeared to be associated with poorer OS [88]. The relationship between higher baseline sPD-L1 levels and shorter OS had been revealed in many other types of cancers, including esophageal cancer, [89, 90], GC, [22, 62, 91, 92, 93, 94] HCC, [72, 95, 96, 97, 98, 99, 100] biliary tract cancer, [101] RCC, [18, 102] upper tract urothelial carcinoma, [103] lymphoma, [104–107] OC, [7, 108] CRC, [109, 110] soft tissue sarcoma (STS),[111, 112] glioma, [113, 114] and PDAC [59, 115].

Second, higher baseline sPD-L1 levels could also be a biomarker of poor PFS, DFS, or time to progress in cancer patients [79–81]. For example, preoperative circulating sPD-L1 levels were negatively correlated with recurrence-free survival (RFS) [109] and DFS [110] in the CRC patients. A high level of plasma sPD-L1 could be an independent unfavorable prognostic factor of PFS in the patients with metastatic ccRCC [61]. STS patients with higher sPD-L1 levels from the PEMBROSARC basket study tended to experience shorter PFS [111]. Similar results were observed in other cancers, like LC, [67, 69, 82, 83, 84, 85, 86, 87,116,117] HNC, [88, 118] esophageal cancer, [89, 90] GC, [62, 91, 92, 93, 119] HCC, [72, 95, 96, 97, 99] OC, [7] lymphoma, [104–106] glioma, [114] and PDAC [115].

Third, cancer patients with poorer response to treatments tended to have higher baseline sPD-L1 levels than those who had ideal response. For instance, the serum levels of sPD-L1 were significantly higher in ICIs non-responsive HNC patients than that in the responders [100]. In a cohort of esophageal cancer patients treated with anti-PD-1/PD-L1 monotherapy, patients with higher baseline sPD-L1 levels displayed a remarkably increased disease control rate versus that of the lower subgroup [90]. As for the metastatic RCC patients treated with PD-1 inhibitor nivolumab, however, higher baseline sPD-L1 levels were correlated to higher rate of progressive disease [102, 120]. For chemotherapy-treated patients with lymphoma, both lower basal sPD-L1 levels [25, 106] and the reduction of sPD-L1 levels after treatment [107] were associated with higher response rate. Patients with LC,[87, 116, 121, 122] or other solid tumors [80, 90, 123] who have higher baseline sPD-L1 levels also tend to experience adverse clinical response. Meanwhile, sPD-L1 levels could also be used as a risk biomarker for the occurrence of cancer metastasis in patients with CRC, [16, 124] upper tract urothelial carcinoma, [103] STS, [112] NPC, [88] and ccRCC [18].

However, a few studies indicated that higher baseline sPD-L1 levels were associated with the better treatment response or the longer PFS and OS in patients with cancer, such as lymphoma, [27] metastatic ccRCC, [74] and NSCLC [125].

In addition to baseline levels, dynamic changes of sPD-L1 levels during treatment were also reported associating with the prognosis of multiple cancers. In general, the reduction of sPD-L1 levels during treatment was predictive of better prognosis for a variety of cancers, [126] including GC, [91] metastatic ccRCC, [120] biliary tract cancer, [101] TNBC, [65] lymphoma,[26, 107, 127] pancreatic cancer, [115] CRC, [109] and NSCLC, [67] regardless to treatment modalities. However, other studies suggested that the decrease of sPD-L1 levels was associated with the poor prognosis in patients with LC, [21, 69] or mesothelioma [31].

Interestingly, sPD-L1 could also be combined with other biomarkers to enhance the accuracy of prognosis prediction in cancer. For instance, the combinations of sPD-L1 with PD-L1 in tumor cells [128] or PD-L1 positivity in tumor tissues [18] were more beneficial in assessing the postoperative prognosis and the OS of patients with NSCLC or ccRCC. sPD-L1 could also be combined with sPD-1,[69, 125, 129] sCTLA-4, [110] Epstein-Barr virus DNA, [88] CCL5, [90] and Glasgow prognostic score [62] to better predict cancer outcomes.

Therefore, sPD-L1 is a promising biomarker in predicting outcomes and treatment responses in cancer patients, though more prospective, independent validated studies are still warranted.

#### Soluble PD-L2

PD-L2 was another substantial ligand of PD-1, whose clinical significance remains obscure. Soluble PD-L2 was reported in several studies as prognostic biomarker in multiple cancers.

Higher baseline sPD-L2 levels were associated with the better clinical response to dendritic cell vaccine therapy in patients with advanced melanoma [130]. It was also associated with the higher risk of biochemical recurrence and progression in PCa patients [55]. A multicenter study revealed a significant positive correlation between baseline sPD-L2 levels and the occurrence of immune-related adverse events (irAEs) in cancer patients receiving immunotherapy [131].

Increased levels of sPD-L2 were significantly associated with higher risk of recurrence in patients with ccRCC [47] (HR = 2.51, 95%CI: 1.46–4.34) and higher risk of invasive disease in a cohort of NSCLC [132] patients (OR = 4.23, 95% CI: 1.20–17.70). And when combined with other variables like sCD27, the prediction performance of sPD-L2 was greatly improved [132].

### The CD28 family of receptors

#### Soluble CTLA-4 and CD28

sCTLA-4 and sCD28 could be prognostic predictors for multiple cancers. Higher levels of baseline sCTLA-4 were associated with the shorter PFS in patients with cHL (HR = 4.30, 95%CI: 1.54–13.26) [133] or glioma (HR = 2.52, 95%CI: 1.01–6.28) [134]. Another cohort study suggested that both sCD28 and sCTLA-4 levels were predictors of biochemical recurrence in the PCa patients [55]. Similarly, higher sCTLA-4 levels at baseline were also significantly associated with the worse OS, DFS or disease progression in patients with GC, [62] CRC, [110] or HNC [135]. Besides baseline levels, dynamic changes of sCTLA-4 and sCD28 were also found associating with OS in the patients with HBV-related advanced HCC in a multicenter study [136]. Interestingly, for the HCC patients treated with radiofrequency ablation, higher baseline sCTLA-4 levels were linked to the shorter DFS of local recurrence (HR = 2.43, 95%CI: 1.03–5.75) but longer RFS of intrahepatic metastasis (HR = 0.19, 95%CI: 0.05–0.81), which showed the dual roles of sCTLA-4 in immune responses. And this performance of sCTLA-4 could be improved when combined with baseline alpha-fetoprotein levels [137].

#### Other members of the CD28 family

Besides sCTLA-4 and sCD28, soluble forms of other CD28 family members could also be served as biomarkers for cancer outcomes.

In a cohort of solid tumor treated with ICIs, researchers found that the patients with higher levels of baseline sBTLA had worse OS [138]. Likewise, PCa patients with higher baseline sBTLA levels had the higher risk of progression [55]. Similar correlations were also demonstrated in the patients with PDAC, [59] chronic lymphocytic leukemia, [139] ccRCC, [47] and advanced HCC [136, 140]. A multicenter observational study of 81 NSCLC patients [141] showed that elevated sICOS levels during treatment were linked to the improved OS and PFS.

### The B7 family of ligands

As ligands of the CD28 family, the B7 family proteins play a crucial role in regulating T cell activation and tolerance through co-stimulatory and co-inhibitory pathways, thereby extensively involve in tumor immune evasion. Their soluble forms could be promising predictive factors of cancer outcomes.

Higher baseline levels of sCD80 were associated with the worse OS and PFS in patients with STS, [142] NSCLC, [143] and PCa [55]. In addition, studies showed that dynamic changes of sCD80 during treatment were associated with the OS of patients with HBV-related advanced HCC, [136] and the risk of invasive disease of NSCLC [132]. Higher level of sCD86 could be an independent predictor of poorer OS in the patients with multiple myeloma [144]. And both higher levels of sB7-H3 and sB7-H4 at baseline were found to be associated with the better OS (sB7-H3: HR = 0.33, 95%CI: 0.14–0.78; sB7-H4: HR = 0.42, 95%CI: 0.19–0.94) and PFS (sB7-H3: HR = 0.32, 95%CI: 0.17–0.64; sB7-H4: HR = 0.32, 95%CI: 0.16–0.64) in the patients with NSCLC [117].

### Soluble LAG-3

Baseline sLAG-3 levels are associated with patients’ outcomes in multiple cancers, and dynamic changes of sLAG-3 levels could be applied in disease monitoring.

Baseline sLAG-3 levels were associated with poor response to immunotherapy in the patients of advanced PDAC, [60] and melanoma [130]. Moreover, studies showed that the increase of sLAG-3 during treatment might predict the worse OS and the clinical responses of patients with HBV-related advanced HCC treated with icariin, [136] and the patients with locally advanced cervical cancer after concurrent chemoradiotherapy [145]. Also, a significant positive correlation between basal circulating levels of sLAG-3 and the occurrence of irAEs in cancer patients receiving immunotherapy was reported in a multicenter study [131].

### Soluble TIM-3

sTIM-3 could also be a biomarker for cancer outcomes. Higher baseline sTIM-3 levels were associated with higher recurrence risk of the ccRCC patients [47] and worse OS of the PDAC patients [146]. Despite this, changes of sTIM-3 levels during treatment could also be an unfavorable sign of the OS in patients with HCC [136] or the development of relapses to chimeric antigen receptor T-cell therapy in patients with mantle cell lymphoma (MCL) [127].

### The TNF superfamily

Both soluble TNF receptors and ligands were reported as biomarkers of cancer outcomes and adverse reactions to cancer treatments.

For the patients with advanced HCC, dynamic changes of sTNF-α receptor I during Lenvatinib treatment were associated with the response to Lenvatinib treatment [147]. Elevated levels of baseline and post-treatment sTNF-R1 and sTNF-R2 were correlated with decreased OS in the patients with advanced urothelial carcinoma who treated with ICIs [148]. Higher levels of s4-1BB at baseline could also predict the poorer OS in patients with metastatic uveal melanoma [149] and the occurrence of irAEs in other type of cancers [131]. Baseline levels of s4-1BB might predict the risk of MCL patients’ recurrence [127] and the aggressiveness of NSCLC, [132] as well as the clinical response to 4-1BB agonist therapy [150].

Increased sCD27 levels were significantly associated with the higher risk of invasive disease in a NSCLC cohort [132]. In contrast, another study indicated that higher levels of sCD27 after ICI therapy could predict clinical benefit in the patients with advanced solid tumors [151]. Higher baseline levels of sHVEM might also indicate the higher risk of biochemical recurrence and progression in PCa patients [55]. In addition, higher baseline levels of sOX40, [152] sCD30, [148] sCD40, [153, 154] and sGITR [55] were associated with worse prognosis in cancer patients.

Being a soluble form of dual immune checkpoint HVEM, the basal circulating levels of sHVEM were positively correlated with the toxicity of irAEs for cancer patients receiving immunotherapy [131]. A multicenter study revealed a significant positive correlation between baseline sCD27 levels and the occurrence of irAEs [131].

On the other hand, as for the soluble forms of the TNF ligands, lower sCD95L levels in the OC patients could be independent poor prognostic factors for the risk of recurrence (HR = 2.63, 95% CI: 1.16–5.95) [155]. And higher sCD70 levels at baseline were found to be associated with better response and PFS in the NSCLC patients [68]. However, higher levels of sCD254 might be a marker of worse clinical response in the metastatic RCC patients treated with nivolumab [156].

### Other soluble immune checkpoints

Other soluble immune checkpoints were also reported associating with cancer outcomes by researchers. For instance, higher soluble intercellular adhesion molecule 1 (sICAM-1) levels were associated with better PFS and OS in many types of cancers [123]. Despite this, higher baseline sICAM-1 levels could predict worse tumor-free survival in the HCC patients treated with radical hepatectomy, especially when combined with alpha-fetoprotein indicators [157].

In addition, although under-studied, higher baseline levels of many other soluble immune checkpoints including sIDO, [55, 60] sMIC-A,[57, 158] sCD8, [159] sCD73,[160, 161] sCD163, [148] and soluble urokinase plasminogen activator receptor [162] were found to be associated with poor prognosis in the patients with various types of cancers. Furthermore, Yoshida et al. found that an increase in sCD226 during chemotherapy might predict worse treatment response in the patients with esophageal cancer [163].

### Signatures of soluble immune checkpoints

Interestingly, there are studies on solid tumors,[151, 164] locally advanced rectal cancer, [165] and PDAC, [40] focusing on the integration of multiple soluble immune checkpoints as composite signature. And these comprehensive predictive models tended to have a higher predictive value than a single molecule.

In summary, we summarized the role of some crucial soluble immune checkpoint molecules in cancer prognosis prediction (Table 2).

**Table 2:** The role of soluble immune checkpoint molecules in cancer prognosis prediction

Soluble receptor/ligand	Prognosis prediction	Outcomes/prognosis
sPD-1	-	Higher baseline levels associated with the poorer prognosis in patients with OC,[7] TNBC,[11] CRC,[18] ccRCC,[19, 64] PCa,[58] diffuse large B cell lymphoma,[61] PDAC,[62, 63] GC,[65] melanoma,[66] and HCC [67].
	+	Higher baseline levels associated with the better prognosis in patients with GC,[73] advanced HNC,[70] NSCLC,[69–71] NPC,[72] HCC,[74] melanoma,[75] and ccRCC [76].
sPD-L1	-	Higher baseline levels associated with the poorer prognosis in patients with OC,[7, 110] RCC,[19, 104] GC,[33, 65, 93–96, 121] lymphoma,[25, 106–109] mesothelioma,[31] PDAC,[62, 117] metastatic ccRCC,[64, 104, 122] LC,[69, 71, 84–89, 118, 119, 123, 124] HCC,[74, 97–102] NPC,^90^] HNC,[90, 102, 120] esophageal cancer, [91, 92] biliary tract cancer,[103] upper tract urothelial carcinoma,[105] CRC,[111, 112] STS, [113, 114] and glioma [115, 116].
	+	Higher baseline levels associated with the better survival or treatment response in patients with lymphoma,[27] ccRCC,[76] and NSCLC [127].
	-	The reduction of levels during treatment was predictive of the better prognosis for TNBC,[11] lymphoma,[26, 109, 129] NSCLC,[69] GC,[93] biliary tract cancer,[103] CRC,[111] PDAC,[117] and ccRCC [122].
	+	The decrease of levels during treatment associated with the poorer prognosis in patients with LC, [21, 71] and mesothelioma [31].
sPD-L2	-	Increased levels associated with the poorer prognosis in ccRCC [44] patients and NSCLC patients [134].
	+	Higher baseline levels associated with the better clinical response in advanced melanoma patients, [132] and PCa[58] patients.
sCTLA-4	-	Higher levels of sCTLA4 were associated with the shorter PFS in patients with cHL [135] and glioma [136] and the worse prognosis in patients with PCa, [58] GC,[65] CRC,[112] or NC [137].
sCD28	-	Higher level was a risk predictor of biochemical recurrence in the PCa patients [58].
sBTLA	-	Higher levels associated with the worse prognosis in patients with ccRCC, [44] PCa,[58] PDAC,[62] advanced HCC,[138, 142] and chronic lymphocytic leukemia [141].
sICOS	+	Elevated levels during treatment linked to the better OS and PFS in NSCLC patients [143].
B7 ligands	-	Higher baseline levels of sCD80 associated with the worse OS and PFS in patients with STS, [144] NSCLC,[134, 145] and PCa [58]. Higher sCD86 level was a predictor of the poorer OS in patients with multiple myeloma [146].
	+	Higher baseline levels of sB7-H3 and sB7-H4 associated with the better OS and PFS in NSCLC patients [119].
sLAG-3	-	Higher baseline levels associated with the poorer prognosis in patients with advanced PDAC,[63] or melanoma [132]. The increase of levels during treatment associated with the worse prognosis in patients with advanced HCC [138] or locally advanced cervical cancer [147].
sTIM-3	-	Higher levels associated with the worse prognosis in patients with ccRCC, [44] or PDAC [47]. The increase of levels after treatment associated with worse prognosis in patients with advanced HCC,[138] and MCL [129].
s4-1BB	-	Higher baseline levels predicted the poor prognosis in patients with metastatic uveal melanoma,[150] and the risk of MCL patients’ recurrence, [129] the aggressiveness of NSCLC [134].
sCD27	-	Higher baseline levels associated with the poor prognosis in patients with HBV-related HCC [138] or metastatic uveal melanoma [150]. Increased levels associated with the higher risk of invasiveness in NSCLC patients [134].
	+	Higher levels after ICIs therapy predicted clinical benefit in the patients with advanced solid tumors [152].
sHVEM	-	Higher baseline levels indicated the higher risk of biochemical recurrence and progression in PCa patients [58].

Some well-studied soluble immune checkpoints are listed in the above table. + means higher levels of soluble immune checkpoints associated with the poorer prognosis; - means higher levels of soluble immune checkpoints associated with the better prognosis. ccRCC, clear cell renal cell carcinoma; cHL, classical Hodgkin lymphoma; CRC, colorectal cancer; GC, gastric cancer; HCC, hepatocellular carcinoma; HNC, head and neck cancer; ICIs, immune checkpoint inhibitors; LC, lung cancer; MCL, mantle cell lymphoma; NPC, nasopharyngeal carcinoma; NSCLC, non-small cell lung cancer; OC, ovarian cancer; OS, overall survival; PCa, prostate cancer; PDAC, pancreatic ductal adenocarcinoma; PFS, progression-free survival; RCC, renal cell carcinoma; STS, soft tissue sarcoma; TNBC, triple-negative breast cancer

## Therapeutic applications of soluble immune checkpoint molecules in cancer

We illustrated the successful applications of soluble immune checkpoints as biomarkers of cancer outcomes and therapeutic responses in multiple cancers. Further, soluble immune checkpoints could also serve as treatment targets or therapeutic modalities in cancer patients.

### The potential therapeutic value of soluble immune checkpoints

On the one hand, soluble immune checkpoints can be potential therapeutic targets. A study revealed that the CRC patients who had scarce tumor-infiltrating lymphocytes (TILs) in tumor had significantly higher sOX40 levels compared to the patients with TILs, suggesting that targeting sOX40 might hold promise for immunotherapy [166]. Likewise, a recent study demonstrated that targeting sMIC alongside non-blocking antibodies could provide dual co-stimulation to antigen-specific CD8^+^ T cells through NKG2D and CD28, thereby improving the anti-tumor immunity [167]. Subsequently, researchers demonstrated combining anti-PD-L1 ICIs with antibody targeting sMIC significantly improved the survival rate of mice compared to monotherapy, suggesting potential therapeutic implications for patients with MIC^+^/sMIC^+^ metastatic melanoma [168].

On the other hand, changing the levels of soluble immune checkpoints and blocking the interactions between soluble immune checkpint proteins and membrane receptors or ligands have potential therapeutic values for cancers. For example, therapeutic plasma exchange in the melanoma patients could enhance the efficacy of immunotherapy by reducing the levels of sPD-L1 and extracellular vesicles PD-L1 [169, 170]. Moreover, a recent study demonstrated that the small molecule inhibitors CH-4 and its analogue CH-4.7 could effectively inhibit the PD-1/sPD-L1 interaction, thereby enhancing anti-tumor immunity in the T cell acute lymphoblastic leukemia model [171]. Similarly, the vaccinia virus M2 protein, capable of binding to CD80/CD86 and inhibiting their interactions with soluble CD28/CTLA-4, while promoting the binding of sPD-L1 and sCD80, exhibited potential as a novel immunosuppressive agent [172].

### Soluble immune checkpoints as therapeutic modalities

#### Monotherapy

Soluble immune checkpoints may serve a similar function to membrane antibodies, and are therefore anticipated to be utilized in the treatment of cancer. For example, sPD-1 demonstrates a functional efficiency comparable to that of anti-PD-1 or anti-PD-L1 monoclonal antibodies (mAb), interfering the interaction between PD-L1 or PD-L2 ligands and their cognate receptor, membrane-bound PD-1 (mPD-1) on the surface of T lymphocytes. Therefore, sPD-1 could serve as an alternative “antibody” to mAb-based immunotherapy and promised preferable anti-tumor immune effects in OC [173] and BC [75] models. In addition, a study revealed that L3C7c, a high-affinity variant of human sPD-L1, could improve the ability of T cells to inhibit melanoma growth and showed promise as a new-generation tumor immunotherapy agent based on PD-1/PD-L1 axis blockade [174]. Similarly, sCD80 could also increase tumor-infiltrating T cells and significantly prolong the survival time of tumor-bearing mice [175]. Targeting alternative splicing also has the potential to be a novel cancer immunotherapy. Inhibiting serine arginine-rich splicing factor (SRSF1 and SRSF3) could regulate alternative splicing of PD-1 to generate sPD-1, thereby preventing T cell exhaustion [176, 177]. In conclusion, soluble immune checkpoints might be a novel therapy for cancer treatment.

### Combined therapy

#### Construction of recombinant vector

Oncolytic viruses are an excellent platform for developing effective strategies in cancer immunotherapy. However, several challenges remain in the use of viro-immunotherapy for cancer. Therefore, some researchers combine viruses with soluble immune proteins to efficiently overcome several major hurdles. For example, NDV/Anh-TRAIL, a recombinant Newcastle disease virus (NDV) Anhinga strain capable of secreting soluble TNF-related apoptosis-inducing ligand (TRAIL), showed potential as a candidate drug for glioma treatment [178]. In China, Wei and his colleagues generated a recombinant adenovirus expressing a soluble fusion protein, sPD1/CD137L, which was effective in suppressing tumor growth and improving survival in the HCC mouse model [179].

Furthermore, soluble recombinant 4-1BBL protein generated by fusing the extracellular domains of murine 4-1BBL to a modified version of streptavidin, could inhibit the development of lung tumors induced by tobacco carcinogens in mice [180]. Similarly, a recombinant vector pMCSG7-hsTNF-R2 was constructed to generate human soluble TNF-R2 recombinant protein, which was expected to be used as an immunotherapy drug for TNF-R2^+^ cancer in an in vitro bioactivity evaluation [181].

#### Combined with other therapeutic strategies

First, several challenges remain in the use of immunotherapy for cancer, such as poor immune cell infiltration, insufficient co-activation signals, and negative regulation of immune checkpoints. Combine soluble immune checkpoints with immunotherapy might improve anti-tumor immunity. Recent studies mostly focused on combination with CAR T-cell immunotherapy. For example, Zhang et al. established modified CAR-T cells called sPD-1 CAR-T cells, which could secrete sPD-1 and had higher cytotoxicity against CD19^+^ PD-L1^+^ tumor cells in vitro compared with conventional CAR-T cells. The sPD-1 CAR-T cells could effectively reduce tumor burden and prolong the survival time of mice [182]. Similarly, researchers of another study engineered CAR T cells to secrete the soluble trimeric 4-1BBL fused to anti-PD-1 single-chain fragment variable region (αPD1-41BBL), and the CAR19.αPD1-41BBL T cell-treated mice displayed significant improved tumor growth control and OS [183]. Also, Xia et al. designed HER2-specific sPD-1-CAR-NK cells for BC treatment. These bio-engineered NK cells could transport sPD-1 specifically into cancer cells with high HER2 expression, thereby enhancing the anti-tumor effect of HER2-CAR-NK cells [184].

Second, soluble immune checkpoints could also be combined with other therapeutic strategies. In a study combined sPD-1-mediated immune checkpoint therapy with chlorin e6-assisted sonodynamic therapy, Tan et al. generated nanobubbles loaded simultaneously with sPD-1 and chlorin e6. Compared with monotherapy, the combined therapy showed the best immunotherapy effect on HCC [185]. Besides, targeting alternative splicing combined with adoptive cellular immunotherapy could enhance the levels of sPD-1 and reverse T cell exhaustion by disrupting mPD-1/PD-L1 interaction in effector T cells [186].

The above treatments were mostly tested in mice or cell lines. Encouragingly, there are already human clinical trials exploring the safety and efficacy of soluble immune checkpoints in combination with other therapies. Researchers in a study combined sLAG-3 with the PD-1 antagonist pembrolizumab to treat patients with metastatic melanoma and the results showed strong antitumor activity [187]. Later, Hans et al. combined sLAG-3 with paclitaxel in a treatment for metastatic HR^+^ BC patients and displayed a numerically improvement in OS, though not statistically significant [188].

Overall, we also summarized the applications of some crucial soluble immune checkpoint molecules in cancer treatment (Table 3).

**Table 3:** The applications of soluble immune checkpoint molecules in cancer treatment

Soluble receptor/ligand	The role in cancer treatment
sPD-1	• sPD-1 could serve as an alternative “antibody” to mAb-based immunotherapy [77, 173] • sPD-1 could also be combined with sonodynamic therapy,[185] CAR-T, [182] and CAR-NK cells therapy [184].
sPD-L1	• CH-4 and its analogue CH-4.7, [171] therapeutic plasma exchange, [169, 170] and L3C7c[174] could effectively interfere the PD-1/sPD-L1 interaction, leading to anti-tumor immunity.
sCD80 & sCD86	• sCD80 could increase tumor-infiltrating T cells and significantly prolong the survival time of tumor-bearing mice [175]. • The Vaccinia virus M2 protein binding to CD80/CD86, exhibited the potential as a novel immunosuppressive agent [172].
sMIC	• Targeting sMIC could improve anti-tumor immunity [167]. • Combining with anti-PD-L1 ICIs suggested potential therapeutic implications for the patients with MIC^+^/sMIC^+^ metastatic melanoma [168].
Soluble TNF	• Targeting sOX40 might hold promise for immunotherapy in CRC [166]. • Soluble recombinant 4-1BBL protein was shown to inhibit the development of lung tumors [180]. • Human soluble TNF-R2 recombinant protein was expected to be used as an immunotherapy drug for TNF-R2^+^ cancer [181].
CRC, colorectal cancer.	

## Molecular mechanisms of soluble immune checkpoint molecules in cancer development

**Fig. 2:**
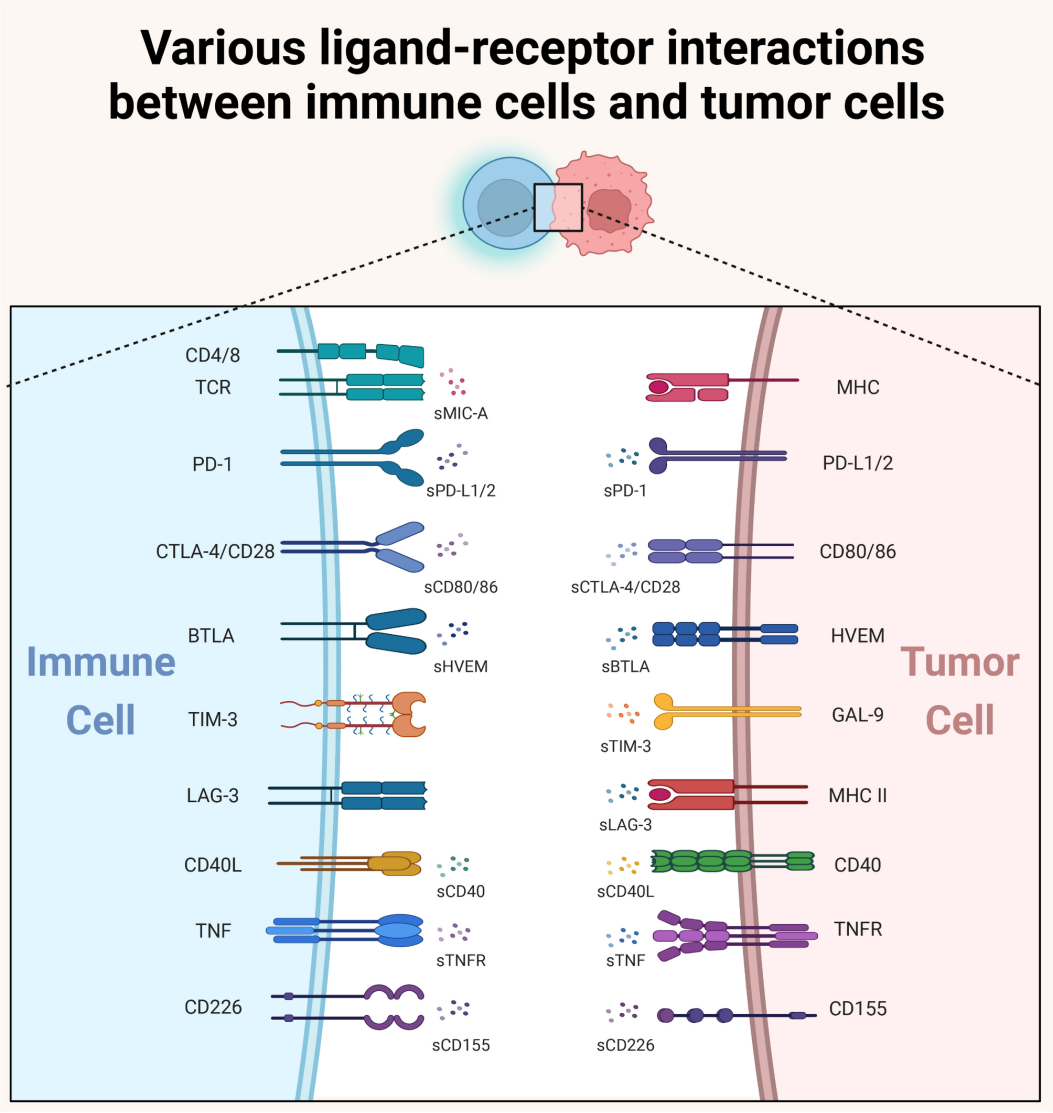
The intricate interaction between soluble immune checkpoints and their membrane-bound receptors / ligands in TME. Soluble immune checkpoints could bind with their receptors or ligands in immune cells or tumor cells, thereby affecting the anti-tumor immunity in TME

Soluble immune checkpoints can be produced by several molecular mechanisms: (1) ectodomains cleaved by proteolysis and excreted to extracellular space by enzyme release, (2) selective mRNA splicing, and (3) released as components of extracellular vesicles.189 These mechanisms prompt them to alter the body’s immunity through a plethora of mechanisms, which have an impact on the development of tumors. The interaction between soluble immune checkpoint molecules and membrane-bound immune checkpoints receptors/ligands in TME could significantly impact anti-tumor immunity and cancer outcomes. To make it clear, we depicted the interactions of mentioned soluble checkpoints and their membrane ligands/receptors in Fig. 2. Elucidating the fundamental mechanisms governing soluble immune checkpoints and their membrane counterparts in cancer could facilitate their utilization in guiding cancer therapeutic strategies.

First, soluble forms of co-suppressive immune checkpoints have different effects on cancer development. On the one hand, they could bind to the corresponding membrane-bound ligands/receptors, thereby hindering the inhibitory effect of membrane-bound ligands/receptors on immune cells, ultimately inhibiting tumor growth. For instance, sPD-1, retaining the function of full-length PD-1, is able to bind to mPD-1 ligands and thereby blocking their interaction with mPD-1 and increasing the effector function of T cells and NK cells [75, 189]. Similarly, sPD-L1 can act as a receptor antagonist, reversing T cell inhibition mediated by mPD-L1 [190]. Also, the soluble form of Siglec-5 (sSiglec-5) was found to intensify the cytotoxicity of T cells to cancer cells [191]. On the other hand, soluble forms of co-suppressive immune checkpoints could also inhibit the function of immune cells, thereby promoting cancer development. For example, in cHL cell lines, sPD-1 could induce PD-L1 reverse signaling, which was associated with inhibition of the mitogen-activated protein kinase (MAPK) pathway and reduced mitochondrial oxygen consumption, thereby promoting tumor growth, proliferation, and metabolism of cHL [14]. sPD-L1 has a similar inhibition to mPD-L1 on T effector cells in in vitro assays, which could induce regulatory B cell differentiation and inhibit peripheral T cells [192–194]. sCTLA-4 was also found to have immunosuppressive abilities like CTLA-4 [195]. Specifically, sCTLA-4 could restrict CD8^+^ T cells to a non-cytotoxic state and attenuate T cell activation, thereby inhibiting anti-tumor immunity and promoting tumor growth [196]. In BCC, sCD200 in TME could inhibit MAPK pathway signaling, resulting in the almost non-existence of tumor-infiltrating NK cells and further promoting tumor development [197].

Second, soluble forms of co-stimulatory immune checkpoints could also play different roles during carcinogenesis. Firstly, they could bind to corresponding membrane-bound ligands/receptors, thereby hindering the membrane-bound ligands/receptors from activating immune cells and ultimately promoting tumor growth. For instance, tumor-derived sMIC-A could bind to membrane-bound NKG2D receptors, thereby blocking the activation of NKG2D pathways, inhibiting the cytotoxicity of NK and T cells against tumor cells [57, 168]. Similarly, sCD160 could also exert immunosuppressive activity by binding to HLA molecules or HVEMs on target cells, thereby inhibiting the cytotoxicity of NK cells [198]. Secondly, soluble forms of co-stimulatory immune checkpoints could also promote the efficacy of immune cells, thereby inhibiting tumor development. For example, sCD80 could maintain T cell activity by simultaneously blocking PD-1 and binding to CD28. The activated T cells could increase the production of IFNγ and IL-2, which in turn boosting anti-tumor immunity via TCR and CD28 signaling [175].

As surface molecules on cancer cells or immune cells, membrane-bound immune checkpoints act through trans or cis interactions to modulate immune responses, depending on factors like expressing cells, relative expression levels, action forms, and downstream cells [199]. For example, trans-interaction of PD-L1 or PD-L2 with PD-1 on T cells can lead to inhibition of signaling, while the cis-interaction of PD-L1-CD80 can play a positive role in anti-tumor immunity. In contrast, soluble immune checkpoints could not only exhibit similar functional effects to membrane-bound immune checkpoints, but also have complicated impacts on the immune system due to their unique forms. Therefore, a comprehensive understanding of the roles of soluble immune checkpoints in TME is conducive to the development of immunotherapy in future.

## Conclusion and prospective

**Fig. 3:**
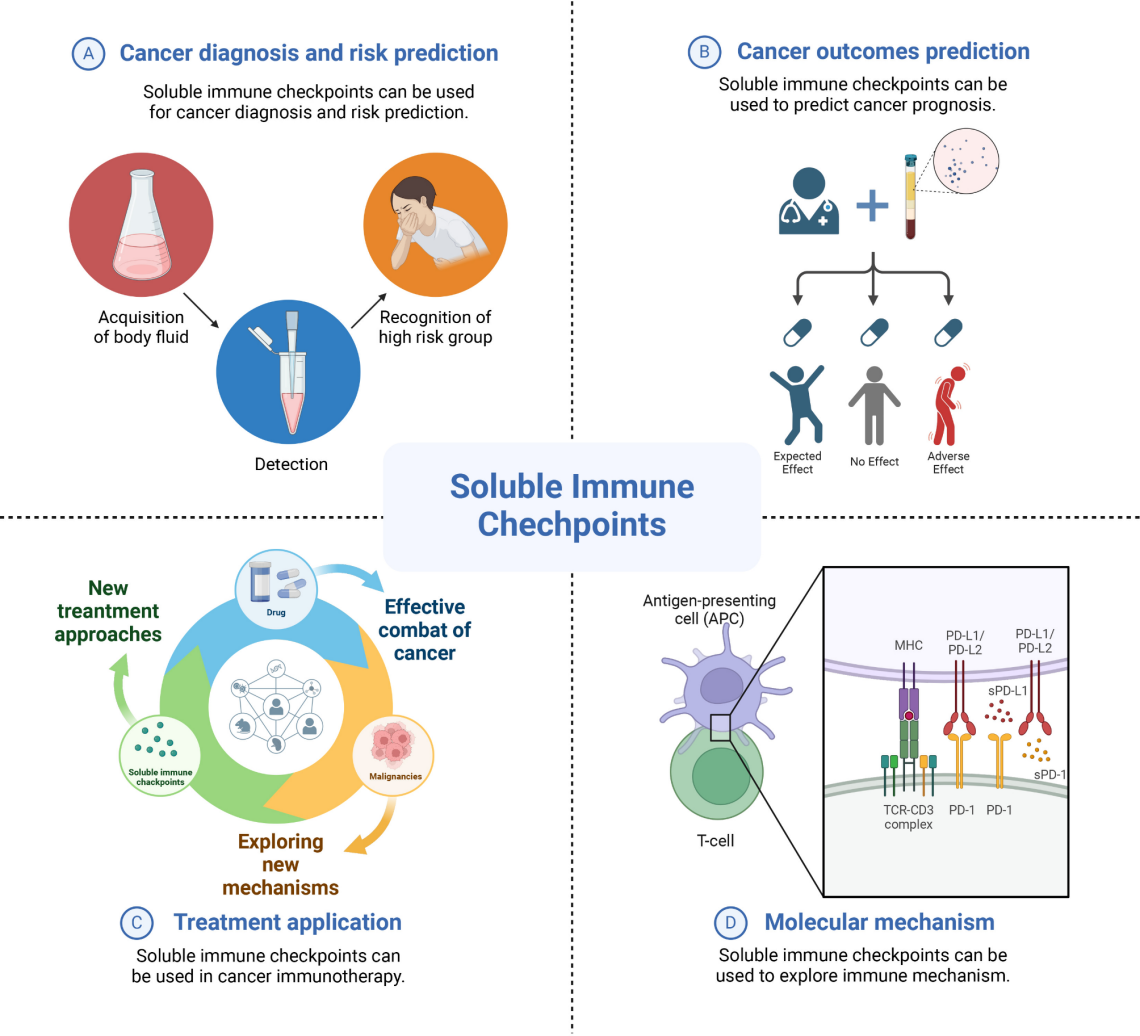
Soluble immune checkpoints in cancer risk prediction, outcomes prediction, therapeutic application, and molecular mechanism. Soluble immune checkpoints could be used as biomarkers for cancer surveillance strategies and targets for checkpoint blockade therapies, while also facilitating cancer immunotherapy and the exploration of immune mechanisms

Soluble immune checkpoint molecules have been a hotspot of research due to their pivotal roles of regulating immune responses in TME. In this review, we systematically reviewed the literatures regarding the major soluble immune checkpoint molecules in cancer screening, outcome prediction, and potential molecular mechanisms. Soluble immune checkpoint molecules could be easily detected in blood and tissues in multiple cancers, and they could be critical factors reflecting the risk of cancer susceptibility, prognosis, and the sensitivity to the treatment. Their interaction with corresponding receptor/ligand in the membrane of cells in TME also indicated potential therapeutic targets and molecular mechanisms (Fig. 3).

Researches on soluble immune checkpoints in cancer are still expanding. sPD-1 and sPD-L1 could be the mainstream biomarkers of immunotherapy as well as the therapeutic targets interfering PD-1/PD-L1 binding in TME, though the molecular mechanisms remain unclear due to complicated splice/cleavage of the proteins. Further studies are also warranted to explore the predictive significance of other soluble immune checkpoints in cancer, like sLAG3 and sTIM3. Soluble immune proteins hold great promise for cancer treatment, either as monotherapy analogous to the function of monoclonal antibodies or in combination with other therapies to enhance overall antitumor activity and provide better treatment for patients. Therefore, more prospective clinical trials are required to provide more evidence of clinical applications of these soluble immune checkpoint molecules. In light of these explorations, we propose that soluble immune checkpoint molecules could be promising biomarkers and targets for cancer patients in the era of precise medicine.

## Data Availability

No datasets were generated or analysed during the current study.

## Abbreviations

BC: Breast cancer
BCC: Basal cell carcinoma
BTLA: B and T lymphocyte attenuator
ccRCC: Clear cell renal cell carcinoma
cHL: Classical Hodgkin lymphoma
CI: Confidence interval
CRC: Colorectal cancer
CTLA-4: Cytotoxic T-lymphocyte antigen-4
DFS: Disease-free survival
GC: Gastric cancer
HCC: Hepatocellular carcinoma
HLA-G: Human leukocyte antigen-G
HNC: Head & neck cancer
HR: Hazard ratio
HVEM: Herpesvirus entry mediator
ICIs: Immune checkpoint inhibitors
irAEs: Immune-related adverse events
LAG-3: Lymphocyte activation gene-3
LC: Lung cancer
mAb: Monoclonal antibodies
MAPK: Mitogen-activated protein kinase
MCL: Mantle cell lymphoma
MHC: Major histocompatibility complex
mPD-1: Membrane-bound PD-1
NAC: Neoadjuvant chemotherapy
NDV: Newcastle disease virus
NK: Natural killer
NPC: Nasopharyngeal carcinoma
NSCLC: Non-small cell lung cancer
OC: Ovarian cancer
OR: Odds ratio
OS: Overall survival
PCa: Prostate cancer
PD-1: Programmed cell death protein 1
PDAC: Pancreatic ductal adenocarcinoma
PD-L1: Programmed cell death protein 1 ligand 1
PFS: Progression-free survival
RCC: Renal cell carcinoma
RFS: Recurrence-free survival
SCLC: small cell lung cancer
sICAM-1: Soluble intercellular adhesion molecule 1
STS: Soft tissue sarcoma
TILs: Tumor-infiltrating lymphocytes
TIM-3: T cell immunoglobulin mucin-3
TME: Tumor microenvironment
TNBC: Triple-negative breast cancer
TNF: Tumor necrosis factor
TRAIL: TNF-related apoptosis-inducing ligand
